# Molecular surveillance of intestinal parasites and *Acanthamoeba* species in soils from outdoor built environments in rural northwestern Argentina

**DOI:** 10.1051/parasite/2026039

**Published:** 2026-07-29

**Authors:** María Cristina Almazán, Melisa Evangelina Díaz-Fernández, Elvia Ester Nieves, Marisa Juárez, Lucia Estela Mejia, Emilio Rey Mejia, Alejandro Javier Krolewiecki, Philip J Cooper, Rubén Oscar Cimino, Rojelio Mejia

**Affiliations:** 1 Instituto de Investigaciones de Enfermedades Tropicales (IIET), Universidad Nacional de Salta (UNSA), Orán Salta Argentina; 2 Consejo Nacional de Investigaciones Científicas y Técnicas (CONICET) Argentina; 3 Cátedra de Química Biológica, Facultad Regional Orán, Universidad Nacional de Salta Salta Argentina; 4 Pediatrics – Tropical Medicine, Baylor College of Medicine Houston Texas United States of America; 5 Robert Turner College and Career High School Pearland Texas United States of America; 6 Fundación Mundo Sano Buenos Aires Argentina; 7 Escuela de Medicina. Universidad Internacional del Ecuador Quito Ecuador; 8 School of Health and Medical Sciences City St George’s University of London London United Kingdom; 9 Cátedra de Química Biológica, Facultad de Ciencias Naturales, Universidad Nacional de Salta Salta Argentina

**Keywords:** Environmental surveillance, Soil-transmitted helminths, Free-living amoebae, qPCR, One Health

## Abstract

Background: Soil-transmitted helminths (STHs) and other enteric parasites remain endemic in rural communities of northwestern Argentina. Environmental surveillance may provide additional information on transmission dynamics and community exposure. Results: We analyzed 116 soil samples collected from 28 sites, including 27 households in Solazuti and one rural school in El Cedral (Orán Department, Salta Province, Argentina), using multi-parallel real-time quantitative PCR targeting intestinal protozoa, STHs, zoonotic nematodes, and *Acanthamoeba* spp. Parasites were detected in outdoor built environments, with 44% of Solazuti households showing at least one positive sample. *Ascaris lumbricoides* and *Toxocara canis* were the most frequently detected helminths and showed distinct spatial patterns. Detection of *A. lumbricoides* was strongly associated with the presence of other parasites (OR 18.11, 95% CI 8.30–38.6; *p* < 0.0001). Additionally, *A. lumbricoides*-positive households harbored significantly more parasite species than *A. lumbricoides*-negative households (*p* = 0.0343), and *A. lumbricoides* frequency correlated with parasite richness (Spearman r = 0.4145, *p* = 0.0316). *Acanthamoeba* spp. was nearly ubiquitous (96.4% of sites), including the school environment, with a 75% contamination rate. Conclusions: Molecular analysis of soil samples revealed spatially heterogeneous but sustained exposure to multiple pathogens in rural outdoor environments. Soil-based qPCR may complement traditional surveillance by providing community-level data independent of human sampling.

## Abstract (fr)

Contexte : Les géohelminthes et autres parasites entériques restent endémiques dans les communautés rurales du nord-ouest de l'Argentine. La surveillance environnementale peut fournir des informations complémentaires sur la dynamique de transmission et l'exposition de la population. Résultats : Nous avons analysé 116 échantillons de sol prélevés sur 28 sites, dont 27 ménages à Solazuti et une école rurale à El Cedral (département d'Orán, province de Salta, Argentine), par PCR quantitative en temps réel multiparallèle ciblant les protozoaires intestinaux, les géohelminthes, les nématodes zoonotiques et les espèces d'*Acanthamoeba*. Des parasites ont été détectés dans les environnements bâtis extérieurs, 44 % des ménages de Solazuti présentant au moins un échantillon positif. *Ascaris lumbricoides* et *Toxocara canis* étaient les helminthes les plus fréquemment détectés et présentaient des profils de distribution spatiale distincts. La détection d'*A. lumbricoides* était fortement associée à la présence d'autres parasites (OR 18,11, IC à 95 % 8,30–38,6 ; p < 0,0001). Les ménages positifs pour *A. lumbricoides* abritaient significativement plus d'espèces parasitaires que les ménages négatifs (p = 0,0343), et la fréquence d'*A. lumbricoides* était corrélée à la richesse parasitaire (Spearman r = 0,4145, p = 0,0316). *Acanthamoeba* spp. était quasi omniprésent (96,4 % des sites), y compris en milieu scolaire, avec un taux de contamination de 75 %. Conclusions : L'analyse moléculaire d'échantillons de sol a révélé une exposition spatialement hétérogène mais soutenue à de multiples pathogènes dans les environnements ruraux extérieurs. La qPCR sur sol pourrait compléter la surveillance traditionnelle en fournissant des données à l'échelle communautaire, indépendamment des prélèvements humains.

## Introduction

Human parasitic infections acquired through environmental exposure represent a significant public health challenge. Global estimates suggest that billions of people are affected by pathogens transmitted via contaminated soil or water, reflecting the persistent burden of diseases related to inadequate sanitation and environmental contamination [28]. In low-resource settings, where access to basic hygiene infrastructure remains limited, environmental transmission plays a central role in sustaining endemic cycles of intestinal parasites.

Millions of people still lack access to safely managed water sources or adequate sanitation, with approximately 15 million people continuing to practice open defecation in regions such as Latin America [26]. These conditions facilitate the continuous spread and environmental persistence of infective stages of intestinal protozoa and helminths, contributing to significant morbidity every year. International public health frameworks emphasize that strengthening water, sanitation, and hygiene (WASH) interventions could substantially reduce exposure to environmental parasites, potentially preventing a significant proportion of the global disease burden [23, 28].

Soil is a fundamental reservoir for the transmission of several human parasites, particularly soil-transmitted helminths (STHs) and other zoonotic nematodes. Many species require the development or maturation of their infectious stages in the soil, making it an active component of parasite life cycles rather than a passive medium. Similarly, environmentally resistant protozoan cysts and oocysts can persist in soil for long periods, allowing for sustained exposure in both human and animal hosts [6, 27]. Therefore, understanding the distribution and persistence of parasitic stages in soil is crucial for assessing risk, transmission dynamics, and the potential impact of environmentally based control strategies.

STH infections are among the most prevalent neglected tropical diseases (NTDs) worldwide, infecting an estimated 1.5 billion people, or nearly 20% of the world’s population. The STH species are *Ascaris lumbricoides*, *Trichuris trichiura*, and the hookworms *Necator americanus* and *Ancylostoma duodenale*; recently, *Strongyloides stercoralis* was formally incorporated into global STH control strategies due to its wide distribution and clinical relevance [27]. Given that the life cycle of these helminths often includes a stage of development or survival in the soil, contaminated environments act as persistent reservoirs, facilitating transmission to humans and animals, underscoring the importance of environmental surveillance together with human diagnosis. This is where molecular techniques such as multi-parallel real-time quantitative PCR (qPCR) have become powerful tools, offering significant advantages over traditional microscopy-based methods by addressing limitations in diagnostic sensitivity, the difficulty of detecting polyparasitism, and the challenges of quantifying parasite load. Furthermore, these techniques have proven highly effective for analyzing environmental samples [17].

In addition to intestinal parasites, free-living amoebae such as *Acanthamoeba* spp. represent an emerging concern in environmental health. These opportunistic protozoa are ubiquitous in soil and water environments [11]. Under specific host conditions, *Acanthamoeba* spp. can cause severe human infections, including *Acanthamoeba keratitis* and, more rarely, granulomatous amoebic encephalitis in immunocompromised individuals [13]. Furthermore, they can act as environmental reservoirs for pathogenic bacteria, increasing the biological hazards associated with contaminated environments [7]. Therefore, monitoring *Acanthamoeba* spp. together with STHs, zoonotic nematodes, and intestinal protozoa may provide a broader assessment of environmental health risks.

This study aimed to characterize the profile and extent of soil contamination by intestinal protozoa, STHs, zoonotic nematodes, and *Acanthamoeba* species in a rural community in Argentina using qPCR-based molecular analysis and to explore the potential of environmental detection for epidemiologic surveillance.

## Materials and methods

### Study area and sample collection

Soil samples were collected in November 2023 from 28 sites across the localities of Solazuti and El Cedral in the Orán Department, Salta Province, northwestern Argentina. Both localities are part of San Ramón de la Nueva Orán (SRNO), the department's main city, and are approximately 30 km apart. SRNO lies approximately 270 km northeast of Salta city and 46 km south of the Bolivian border. A subtropical climate characterizes this region and it belongs to the Yungas phytogeographic region (Supplementary Figure 1).

Most of the sampled sites were located in Solazuti (27 out of 28). These consisted of rural households in proximity to one another. The dwellings were of precarious construction, with dirt floors and pit latrines. From each household, up to 5 soil samples were collected from the following zones: the house entrance, front and back patios, and the perimeter of the latrine. The latrines were limited-service latrines on the Joint Monitoring Programme (JMP) sanitation ladder, located about 2–5 m from the house’s entrance. Permission was obtained from tenants and local Public Health officials.

The remaining site (1 out of 28) was sampled in El Cedral, specifically the local school. Nine samples were collected from the schoolyard, a vegetable garden, and adjacent areas. Both selected localities are located in an area with known endemicity for soil-transmitted helminths (STHs), as supported by previous seroprevalence and coproparasitological studies conducted in the region [2, 20].

After obtaining consent from the homeowners, superficial soil samples were collected by scooping the upper soil layer with a sterile 50 mL conical tube until approximately half full (~20–25 g). All samples were properly labeled and stored at room temperature until further processing (Supplementary Table 1).

### Sample flotation and filtration

To concentrate parasitic stages and improve DNA yield, individual soil samples were subjected to a flotation and filtration procedure before DNA extraction. Each sample was weighed and recorded. For the initial washing step, phosphate-buffered saline (PBS) supplemented with 0.05% Tween 20 (Sigma-Aldrich, St. Louis, MO, USA) was added to each sample to a final volume of 45 mL. Samples were vigorously shaken for 5 min and centrifuged at 500× *g* for 5 min at room temperature. After centrifugation, the supernatant was discarded.

The resulting pellet was resuspended in 10 mL of saturated sugar solution (320 g sucrose dissolved in 600 mL distilled water; specific gravity 1.30) to promote the flotation and concentration of parasite eggs, larvae, and cysts before DNA extraction [10, 14]. Samples were shaken for 5 min and centrifuged again at 500× *g* for 5 min at room temperature. The supernatant containing the floated parasitic stages was filtered; 50-mL syringes were connected to a multichannel filtration manifold, enabling simultaneous processing of up to six samples. Each syringe was fitted with a 25 mm filter holder containing a mixed cellulose ester (MCE) filter with a 3 μm pore size (Merck Millipore, Burlington, MA, USA). The filtration manifold was connected to a vacuum pump, and filtration was performed until the entire volume of supernatant had passed through the membrane. The filters were then retained for subsequent DNA extraction.

### DNA extraction

Genomic DNA was extracted from parasites retained on the MCE filters using an MP FastDNA Spin Kit for Soil (MP Biomedicals, Santa Ana, CA, USA), with slight modifications to optimize the lysis of resistant parasitic stages, including eggs and cysts, as previously described [16]. Briefly, each filter was placed into a Lysing Matrix tube containing a mixture of ceramic, glass, and silica beads. To monitor extraction efficiency and detect potential PCR inhibition, an exogenous internal control DNA sequence was spiked into the lysis buffer during this stage, as previously described [4].

To further promote parasite lysis, samples were subjected to a heat disruption step at 90 °C for 10 min in a dry bath, followed by mechanical disruption by bead beating using a Disruptor Genie (Scientific Industries, Bohemia, NY, USA) at 3,000 rpm for 5 min. Subsequent steps, including DNA binding, washing, and purification, were performed according to the manufacturer’s standard protocol. The purified DNA was finally eluted in 100 μL elution buffer and stored at −20 °C until further molecular analysis.

### Multi-parallel real-time quantitative PCR for pathogen detection and quantification

The extracted DNA was analyzed for the presence of a panel of environmental and intestinal pathogens. The molecular targets included the following intestinal parasites: soil-transmitted helminths: *Ascaris lumbricoides*, *Ancylostoma* species, *Necator americanus*, *Trichuris trichiura*, *Strongyloides stercoralis*; zoonotic nematodes: *Toxocara canis*, *Toxocara cati*; and intestinal protozoa: *Cryptosporidium* species, *Entamoeba histolytica*, *Giardia intestinalis*, and *Blastocystis* species.

Additionally, the presence of the free-living amoeba *Acanthamoeba* spp. was studied. This opportunistic organism was included due to its significant environmental ubiquity and potential to cause severe infections in humans.

Detection and quantification were performed using a multi-parallel real-time quantitative PCR [16]. Each species was targeted in singleplex reactions with a final volume of 7 μL. The reaction mixture consisted of 5 μL TaqMan Fast Advanced Master Mix (Applied Biosystems, Foster City, CA, USA) and forward and reverse primers at a final concentration of 900 nM. For detection, FAM-labeled probes featuring a minor groove binder (MGB) and a non-fluorescent quencher (NFQ) were used at a final concentration of 100 nM, as previously reported [18] (Supplemental Table 2). Two μL of extracted DNA were used as the template.

Known concentrations of parasite-specific plasmid DNA were used as positive controls. Nuclease-free water was used as a non-template control (NTC) in every run.

Amplification was carried out using an Open qPCR thermocycler (Chai Bio, Santa Clara, CA, USA) under the following cycling conditions: initial denaturation at 95 °C for 20 s, followed by 40 cycles of denaturation at 95 °C for 1 s and annealing/extension at 60 °C for 20 s. For all species analyzed, a cycle threshold (Ct) value of ≤38 was established as the cut-off for a positive result, as previously determined [18]. All parasites except for *Trichuris trichiura* were studied in Argentina. For validation, *T. trichiura* DNA was detected on a QS7 Pro Real-Time PCR System (Thermo Fisher Scientific, Waltham, MA, USA) in Houston, Texas. An aliquot (15–35 μL) of each DNA eluate was spotted onto 0.2 μm filter paper (Millipore, Merck KGaA, Darmstadt, Germany), air-dried, and shipped at ambient temperature to Baylor College of Medicine, Houston, TX, USA. Once received, DNA was extracted from the filter papers by overnight elution at room temperature using the same volume of elution buffer (MP Biomedicals, Santa Ana, CA, USA). An internal control was validated by qPCR to confirm that no DNA was lost. Parasite DNA concentrations (fg/μL) (except for *T. trichiura*) were estimated using a comparative Ct method based on a plasmid standard of known DNA concentration [18]. Relative DNA concentration was calculated according to the equation: sample DNA concentration (fg/μL) = standard DNA concentration (fg/μL) × 2^−(sample Ct − standard Ct). For *T. trichiura*, DNA concentrations were calculated from a standard dilution curve, as previously described [18].

### Statistical analysis

A sampling site was defined as contaminated with a given parasite if at least one soil sample from that site tested positive by qPCR. The contamination rate was calculated as the percentage of qPCR-positive samples out of the total number of samples analyzed.

Descriptive statistics were used to report the frequency of parasite species, the occurrence of polyparasitism (multiple parasite species in a single sample), and the distribution of positive samples across different microenvironments (e.g., property entrances, patios, and latrine perimeters).

To determine associations between sampling zones within households and parasite detection, odds ratios (ORs) with 95% confidence intervals were calculated from 2 × 2 contingency tables, and statistical significance was assessed using Fisher’s exact test and the Chi-squared test. Analyses were performed for overall intestinal parasite detection (“any parasite”) and for parasite species with the highest number of positive samples (*Ascaris lumbricoides* and *Toxocara canis*).

Additionally, the association between *A. lumbricoides* detection and the presence of other parasites was evaluated using OR and Fisher’s exact test. Parasite species richness was defined as the total number of different intestinal parasite species detected within a household, excluding *Acanthamoeba* spp. Species richness was compared between *A. lumbricoides*-positive and *A. lumbricoides*-negative households using the Mann–Whitney test. The association between the number of *A. lumbricoides*-positive samples per household and parasite species richness was assessed using Spearman’s rank correlation coefficient. All analyses were performed using GraphPad Prism v9.5.1, and *p*-values <0.05 were considered statistically significant.

## Results

### Sample-level analysis: overall contamination, parasite diversity, and co-occurrence

A total of 116 soil samples were analyzed, with the majority collected in the Solazuti locality (107/116, 92%), while the remaining samples were obtained from El Cedral school. Internal amplification controls (IACs) were successfully amplified in all but 2 of the 116 samples analyzed. Samples lacking IAC amplification were excluded from further analyses.

The overall environmental contamination rate was 19% (22/114), with samples testing positive for at least one parasite species. Notably, all soil samples from El Cedral school (*n* = 9) tested negative for intestinal parasites, whereas *Acanthamoeba* spp. was detected in 8 of 9 samples. Thus, detectable environmental contamination by intestinal parasites was restricted to the domestic settings of Solazuti.

**Figure 1:**
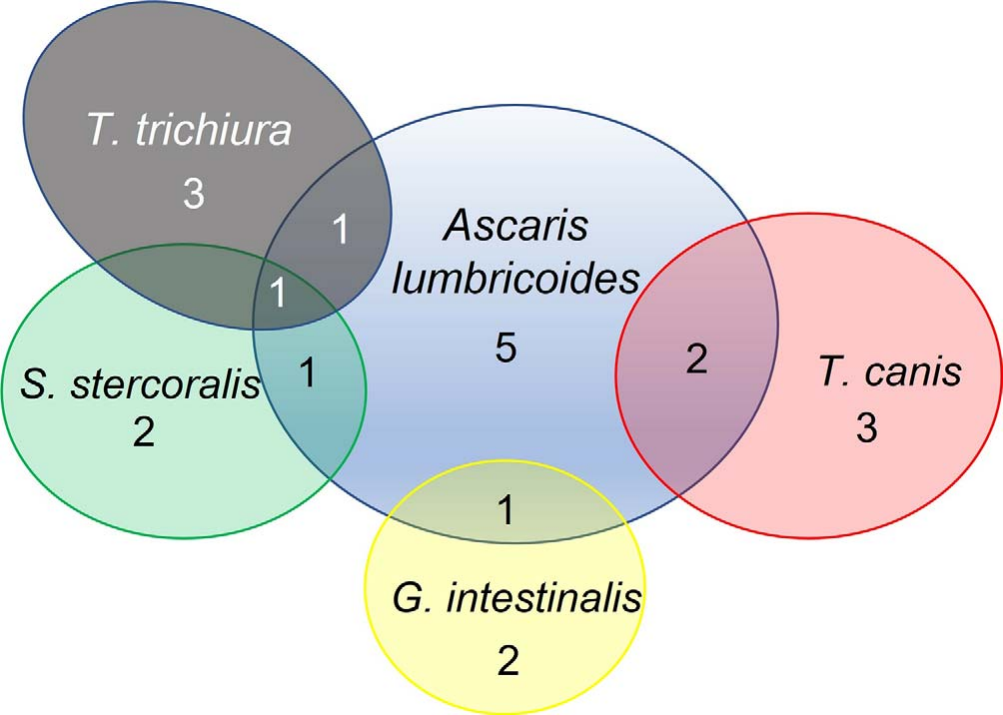
Co-occurrence of parasites. This Venn diagram illustrates the distribution of samples with single and multiple parasitic infections (polyparasitism).

Six different intestinal parasite species were detected across all samples. When analyzed by parasite group, *Ascaris lumbricoides* was the most frequently detected STH, *Toxocara canis* was the most prevalent zoonotic helminth, and *Giardia intestinalis* was the most frequently detected intestinal protozoan (Table 1). Co-occurrence of parasites was observed in 27% (6/22) of the positive samples, representing 5% (6/114) of all analyzed samples. The most frequent combination was *A. lumbricoides* and *T. canis* (Fig. 1).

**Table 1:** Contamination rates (sample-level and site-level) and DNA concentrations of pathogens detected in soil samples from a rural endemic area in northern Argentina.

Parasite/location	Contamination rate of samples	Contamination rate by site	DNA concentration in kg of soil (fg/μL), mean (range)
HELMINTHS			
*Ascaris lumbricoides*			
Overall	9.65% (11/114)	14.28% (4/28)	203.9 (0.099–1494.9)
Solazuti	10.48% (11/105)	14.81% (4/27)	203.9 (0.099–1494.9)
El Cedral school	0% (0/9)	0% (0/1)	0
*Strongyloides stercoralis*			
Overall	3.51% (4/114)	7.14% (2/28)	1.92 (0.019–7.42)
Solazuti	3.81% (4/105)	7.41% (2/27)	1.92 (0.019–7.42)
El Cedral school	0% (0/9)	0% (0/1)	0
*Toxocara canis*			
Overall	4.39% (5/114)	17.86% (5/28)	470591.0 (300.85–2188280.0)
Solazuti	4.76% (5/105)	18.52% (5/27)	470591.0 (300.85–2188280.0)
El Cedral school	0% (0/9)	0% (0/1)	0
*Trichuris trichiura*			
Overall	4.42% (5/113)	14.3% (4/28)	65.13 (25.93–130.15)
Solazuti	4.76% (5/105)	14.81% (4/27)	65.13 (25.93–130.15)
El Cedral school	0% (0/7)	0% (0/1)	0
PROTOZOA			
*Acanthamoeba* species			
Overall	75.23% (82/109)	96.4% (27/28)	9027.2 (251.6–91716.4)
Solazuti	73.3% (74/101)	96.3% (26/28)	8565.8 (6.67–91716.4)
El Cedral school	88.89% (8/9)	100% (1/1)	11722.3 (570.2–65610.1)
*Blastocystis* species			
Overall	0.88% (1/114)	3.57% (1/28)	0.159
Solazuti	0.95% (1/105)	3.70% (1/27)	0.159
El Cedral school	0% (0/9)	0% (0/1)	0
*Giardia intestinalis*			
Overall	2.63% (3/114)	10.71% (3/28)	11.51 (0.125–33.02)
Solazuti	2.86% (3/105)	11.11% (3/27)	11.51 (0.125–33.02)
El Cedral school	0% (0/9)	0% (0/1)	0

### Site-level analysis: household contamination patterns

**Figure 2:**
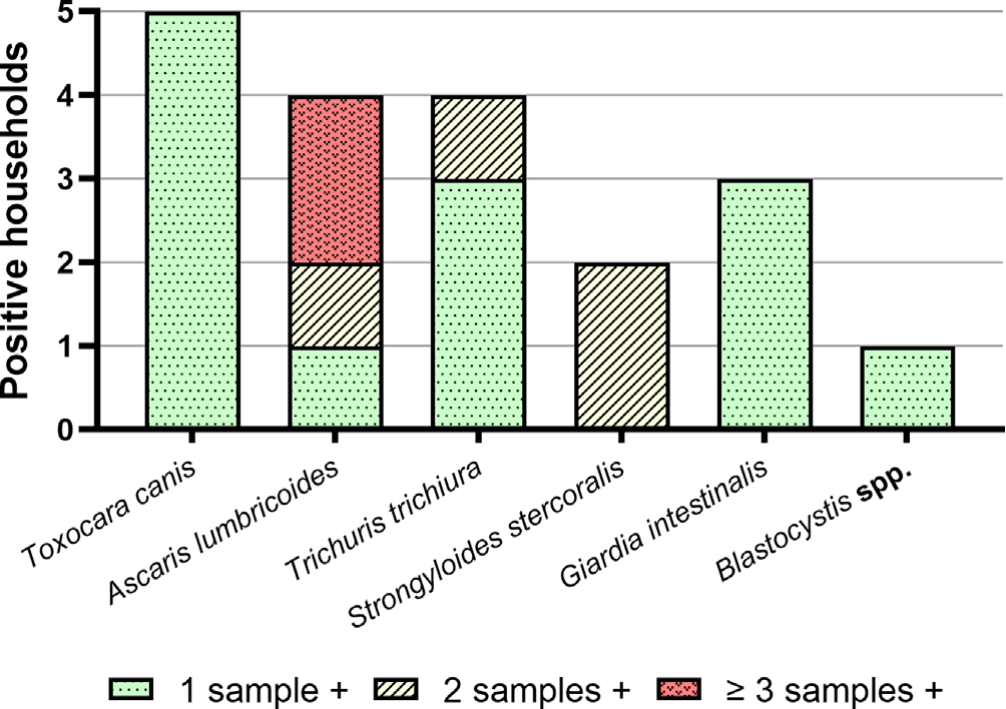
Distribution and extent of environmental contamination in Solazuti households. Bars represent the total number of positive households (*n* = 27) for each detected parasite species. The internal segments of each bar indicate the number of positive samples identified within each household, reflecting the spatial extent of environmental contamination.

At the site level, 28 sampling sites were evaluated, including 27 rural households in Solazuti and the El Cedral school. In Solazuti, environmental contamination was widespread, with 44% (12/27) of all sites testing positive for one or more parasites. Consistent with the sample-level findings, *T. canis* and *A. lumbricoides* were the most frequently detected parasites at the site level (Table 1). Most parasite species showed a focal distribution, with only one positive soil sample per household. In contrast, *A. lumbricoides* and *Strongyloides stercoralis* were often detected in multiple samples (two or more) from the same household, suggesting broader environmental dispersion (Fig. 2).

### Spatial distribution of parasites across household sampling zones

**Figure 3:**
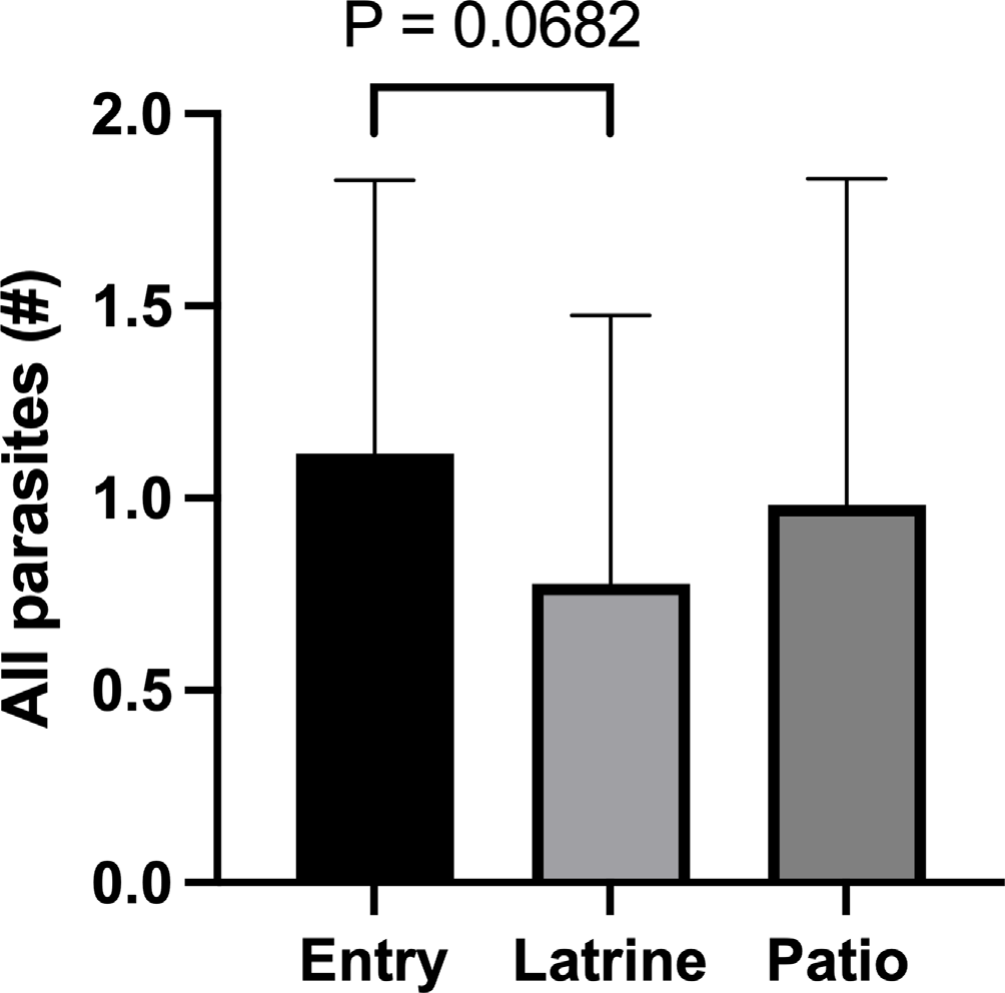
Comparative analysis of parasite species richness across household sampling zones. Mean number of different intestinal parasite species detected per outside-built environment sample. Bars represent the mean diversity observed at house entrances, latrine perimeters, and patio areas, with error bars indicating the standard deviation.

To assess whether parasite detection varied across sampling zones within Solazuti households, odds ratios (ORs) were calculated (Table 2). No statistically significant associations were observed between particular sampling zones (house entrance, latrine, or patios) and the detection of intestinal parasites. Although the house entry point showed slightly higher odds of parasite detection than other household areas (OR = 1.27, 95% CI: 0.48–3.19, *p* = 0.62), the difference was not statistically significant, consistent with a relatively homogeneous distribution of intestinal parasites across household sampling zones. Similarly, the spatial distribution of *A. lumbricoides* did not show a significant association with any specific household zone. In the case of *T. canis*, a marked trend toward higher odds of detection was observed in samples collected at the house entrance (OR = 5.15, 95% CI: 0.98–29.79, *p* = 0.09); however, the estimates were wide and did not reach statistical significance. Furthermore, pairwise comparisons revealed a near-significant trend toward a higher number of parasite species at house entrances compared to latrines (mean 1.16 vs. 0.778, *p* = 0.0682; Fig. 3).

**Table 2:** Association between household sampling zone and soil contamination with intestinal parasites in Solazuti.

Parasite	Entrance	Latrine	Patios	Odds ratio (95% CI)		*P*-Value
Any parasite	8	5	16	Entrance vs. Rest	1.27 (0.48–3.19)	0.62
				Entrance vs. Latrine	1.87 (0.54–6.29)	0.52
				Entrance vs. Patios	1.08 (0.38–3.03)	>0.99
				Latrine vs. Patios	0.58 (0.21–1.88)	0.42
				*Ascaris* + vs. *Ascaris* -	18.11 (8.30–38.60)	<0.0001*
*Ascaris lumbricoides*	3	1	7	Entrance vs. Rest	1.19 (0.32–4.50)	0.73
				Entrance vs. Latrine	0.31 (0.02–2.23)	0.61
				Entrance vs. Patios	1.12 (0.26–4.26)	>0.99
				Latrine vs. Patios	0.27 (0.02–1.73)	0.43
						
*Toxocara canis*	3	1	1	Entrance vs. Rest	5.15 (0.98–29.79)	0.09
				Entrance vs. Latrine	0.31 (0.02–2.23)	0.61
				Entrance vs. Patios	7.04 (0.98–92.80)	0.09
				Latrine vs. Patios	2.16 (0.11–41.68)	0.54

*Footnote*: CI: confidence interval. The “Patios” category combines front and back patios. “Rest” combines Latrine and Patios. *P*-values were calculated using Fisher’s exact test. *Acanthamoeba* spp. were excluded from this analysis. *Statistically significant.

### *Ascaris lumbricoides* as an environmental marker of polyparasitism

**Figure 4:**
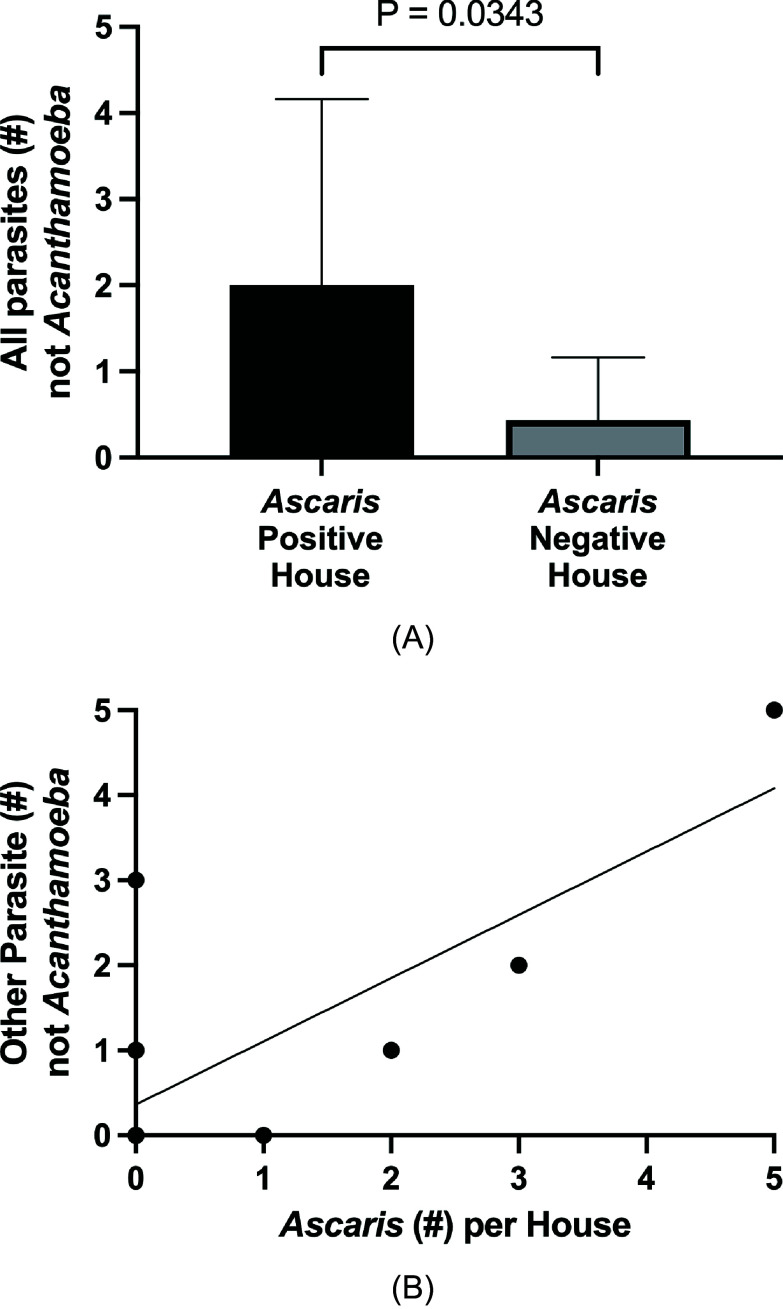
*Ascaris lumbricoides* as an environmental marker for parasite diversity. A) Comparison of intestinal parasite richness (excluding *Acanthamoeba* spp.) between households with and without *Ascaris* contamination. B) Correlation analysis between the frequency of *Ascaris*-positive soil samples per house and the total diversity of other intestinal parasites detected.

Beyond spatial distribution, *A. lumbricoides* contamination was a strong indicator of environmental polyparasitism. Intestinal parasite species richness, ranged from one to four species per household. Among the 12 positive households, eight harbored a single parasite species, whereas four exhibited environmental polyparasitism, with two, three, or four species detected. The detection of *A. lumbricoides* was significantly associated with the presence of other intestinal parasites (OR = 18.11, 95% CI: 8.30–38.6, *p* < 0.0001). Specifically, households contaminated with *A. lumbricoides* harbored a significantly greater intestinal parasite species richness (excluding *Acanthamoeba* spp.) compared to *A. lumbricoides*-negative households (mean 2.0 vs. 0.44, *p* = 0.0343; Fig. 4A). This relationship was further supported by a positive correlation between the number of *A. lumbricoides*-positive soil samples per house and the species richness of other parasites detected (Spearman r = 0.4145, *p* = 0.0316; Fig. 4B).

### Ubiquitous distribution of *Acanthamoeba* spp.

In contrast to the heterogeneous and relatively focal distribution of intestinal parasites, the free-living amoeba *Acanthamoeba* spp. was widely distributed across the study area. It was detected in both Solazuti households and the El Cedral school, with an overall sample positivity rate of 75% (82/109). At the site level, *Acanthamoeba* spp. was identified in 96.4% (27/28) of all sampled locations, with a median of 3 positive samples per site (IQR: 2–4). Notably, in the El Cedral School setting, 8 of 9 samples tested positive.

### Comparison of contamination patterns among detected organisms

**Figure 5:**
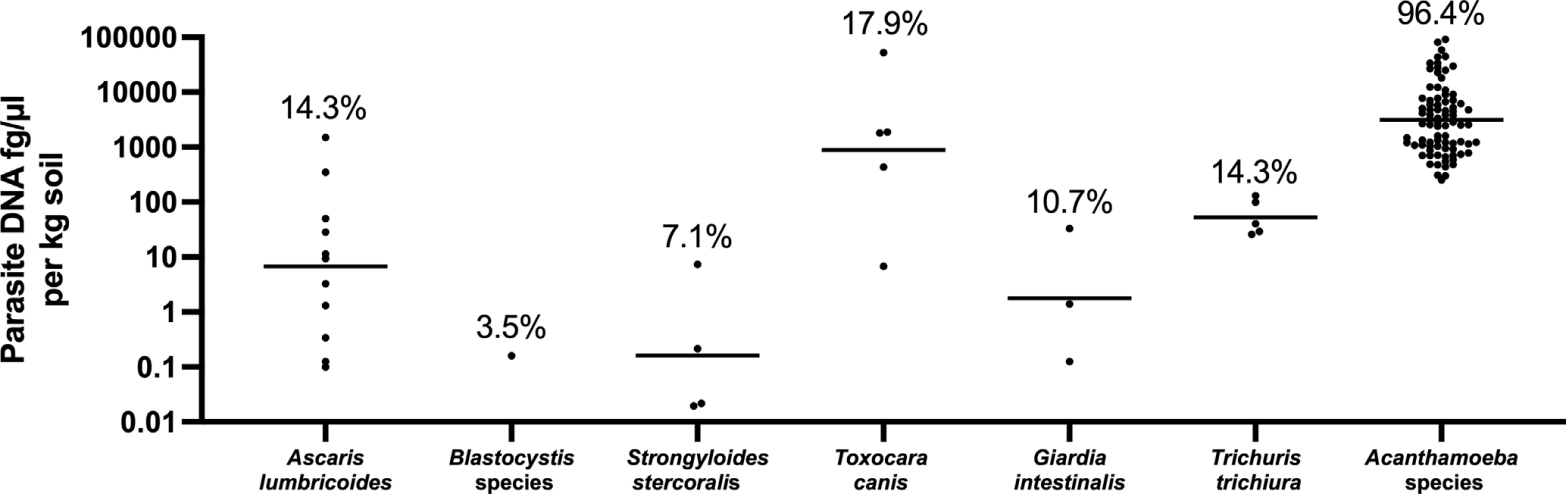
Parasitic load quantification by species. Points represent individual samples, and horizontal lines indicate the median burden. Data are presented on a logarithmic scale. For each species, the percentage of contaminated sites (n = 28) is shown above.

When contamination patterns were evaluated at the site level (*n* = 28), marked differences among pathogen groups emerged. Whereas *Acanthamoeba* spp. was detected in nearly all locations, intestinal parasites were present in a substantially smaller proportion of sites. Of note, *A. lumbricoides* and *T. canis* were identified in 14.3% (4/28) and 17.9% (5/28) of sites, respectively, while other intestinal parasites were detected less frequently (Fig. 5). These findings highlight the distinct environmental distribution patterns of intestinal parasites, in contrast to the near-ubiquitous presence of *Acanthamoeba* spp.

### Parasite DNA load quantification

Finally, to enable cross-sample comparison, DNA load was normalized by the mass of each soil sample, which ranged from 7.1 to 38.4 g (mean ± SD: 23.6 ± 5.7 g). Quantification revealed considerable variations in the environmental DNA burden among the different targets (Table 1). Notably, within all parasites, *T. canis* exhibited markedly higher DNA concentrations than *A. lumbricoides*. Median DNA loads for *T. canis* were approximately two orders of magnitude higher than those observed for *A. lumbricoides* (Fig. 5), despite its lower detection frequency at the sample level.

## Discussion

Environmental surveillance of intestinal parasites provides a complementary perspective for understanding transmission dynamics and persistent exposure risks in endemic settings. In this study, we conducted molecular screening of soil samples from outdoor built environments and from a school in two rural communities in northwestern Argentina, which are known for STH endemicity.

Regarding species composition, *Ascaris lumbricoides* was the most frequently detected helminth, consistent with previous coproparasitological reports from the region [20]. Its predominance is biologically plausible given the high environmental resilience of *Ascaris* eggs [15]. In parallel, the detection of *Strongyloides stercoralis* in peridomestic soil is noteworthy due to its clinical and epidemiologic relevance. Environmental positivity aligns with previous serological and coproparasitological evidence from the area [2, 20] and supports the value of molecular environmental approaches for detecting parasites that are often underdiagnosed using conventional methods.

Among zoonotic helminths, *T. canis* was the only species identified, reinforcing the role of domestic animals in shaping contamination patterns. Although environmental data for the study area are lacking, reports from nearby regions indicate substantial exposure in both humans and dogs [24], supporting the consideration of serological testing in pediatric cases of eosinophilia. *Giardia intestinalis*, a potential zoonotic protozoan that depends on assemblage, was detected at a subset of sites, consistent with previous findings showing marked differences in prevalence across diagnostic methods, highlighting the increased sensitivity of molecular tools [3].

Beyond species composition, spatial distribution revealed distinct ecological patterns. While *A. lumbricoides* and *T. canis* were detected at a similar number of sites in Solazuti, their distribution patterns differed markedly. *Toxocara canis* exhibited a focal, high-intensity pattern, typically restricted to a single sample per household but with the highest DNA concentrations. Its tendency to cluster at household entry points suggests that these areas serve as contamination hotspots, likely reflecting the behavior of dogs that use these zones for rest or transit. Host-related factors, such as high egg-shedding rates in puppies and free-roaming behavior, may further contribute to this localized accumulation [21].

In contrast, *A. lumbricoides* displayed a more dispersed distribution, with multiple positive samples across peridomestic areas within the same household. Lower detection in latrine areas than in patios supports previous observations that contamination is not confined to sanitary infrastructure but is distributed across the peridomestic environment [19]. This pattern likely reflects behavioral factors, including inconsistent latrine use and hygiene practices, particularly among children. Notably, *A. lumbricoides* was significantly associated with intestinal parasite species richness. Households with *A. lumbricoides* contamination showed greater richness of intestinal parasite species, and the number of *A. lumbricoides*-positive samples positively correlated with this richness. Together, these findings indicate that *A. lumbricoides* acts as a reliable environmental marker of persistent parasite life cycles and deficient sanitation.

In contrast to intestinal parasites, *Acanthamoeba* spp. were nearly ubiquitous across all sampled sites, including the school environment, and exhibited consistently high DNA loads. Its detection in the absence of enteric parasites at El Cedral School supports the validity of negative results for intestinal pathogens. Unlike enteric parasites, *Acanthamoeba* is a resilient, free-living amoeba that can serve as a reservoir for pathogenic bacteria, posing additional health risks to children and immunocompromised individuals [7, 13].

These findings highlight the need to interpret environmental contamination within a broader One Health framework that integrates human, animal, and environmental components. For example, *S. stercoralis* illustrates this complexity; its ability to complete part of its life cycle in soil facilitates persistence and reinfection even after treatment [12]. In this context, chemotherapy alone may be insufficient. Previous research has shown that inadequate sanitation nearly quadruples the odds of skin-penetrating helminth infection [5], while integrated interventions that combine treatment with water and sanitation improvements reduce reinfection [25]. The spatial distribution of *A. lumbricoides* further suggests that infection risk depends not only on infrastructure but also on human behavior, underscoring the need for integrated strategies that combine chemotherapy, WASH interventions, health education, veterinary management, and environmental surveillance within a One Health framework.

Several limitations should be acknowledged. Unequal sample sizes across sites limit direct comparisons. The absence of hookworm detection, despite an 8% regional prevalence, suggests that sampling depth and seasonality may have influenced the results, as larvae migrate vertically in response to moisture [9, 22]. Additionally, differences in environmental persistence and recovery efficiency among parasite stages may have introduced species-specific detection biases. Primer specificity must also be considered; the *Ancylostoma* assay may detect zoonotic species beyond *Ancylostoma duodenale* [1]. Notably, *Trichuris* was detected in 14.3% of households despite human prevalence below 4% [3, 20]. NCBI nucleotide BLAST analysis revealed cross-reactivity with *Trichuris vulpis*, a canine parasite. Given the high detection rate of *T. canis*, some *Trichuris* signals may be due to *T. vulpis*, highlighting the need for species-specific markers or alternative molecular targets in future studies to minimize cross-reactivity. Another limitation is that DNA detection does not necessarily indicate the presence of viable parasite stages and may therefore not reflect the true risk of infection. However, this could be addressed by incorporating viability assessment techniques, such as vital dyes or mRNA detection [8]. This limitation remains inherent in molecular environmental analyses despite the use of concentration, flotation, and filtration steps that reduce the amount of non-viable eggs/larvae/cysts. Finally, it is important to note that DNA concentrations (fg/μL per kg of soil) represent relative contamination intensity for each species independently. Due to biological differences (e.g., genome size, copy number, life stage, and extraction efficiency), these values are not directly comparable across different species. Therefore, these data should be interpreted as relative indicators of environmental contamination within each species, rather than absolute measures of parasite burden.

## Conclusions

Soil-based qPCR screening is a valuable complement to traditional coproparasitological diagnosis, offering independence from human sampling, while providing community-level epidemiologic insights. Nevertheless, further research is required to validate its role as a surveillance tool, particularly to determine the extent to which environmental parasite detection correlates with household infection prevalence. Finally, the widespread detection of *Acanthamoeba* spp. across both household and school environments further emphasizes the value of environmental surveillance for identifying biological hazards that may not be captured through intestinal parasite monitoring alone.

## Figures and Tables

**Figure:**
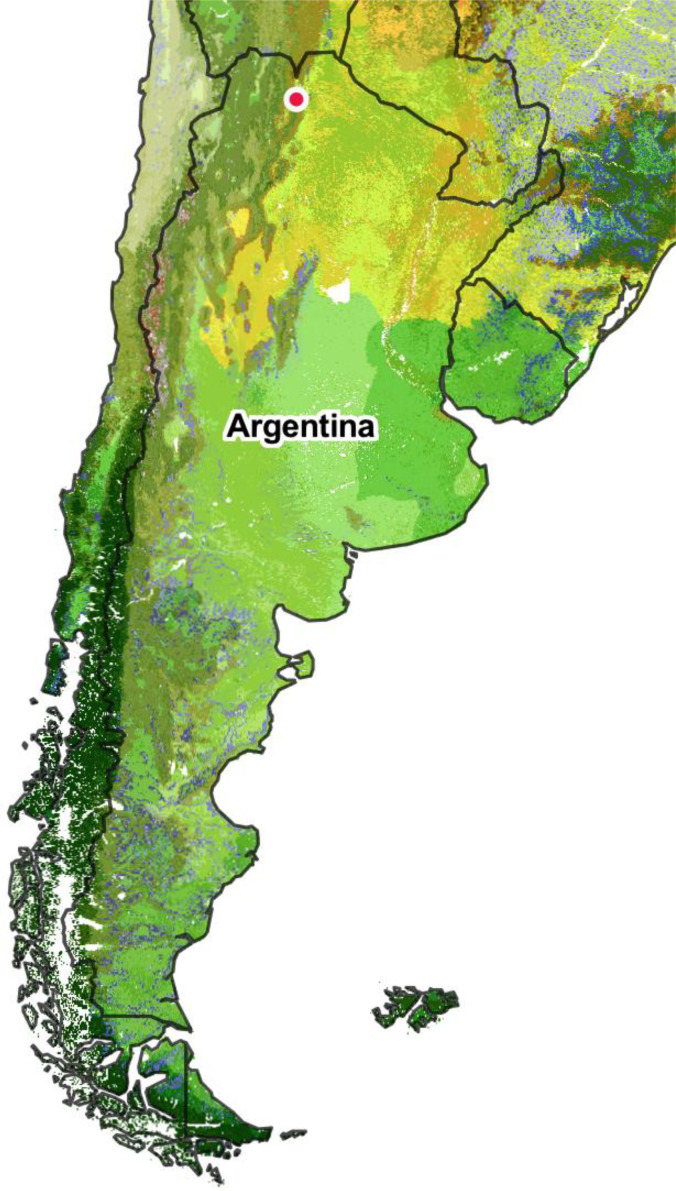
*Supplementary Figure 1*: Ecosystems and regions of Orán Department, Salta Province, Argentina.

## Supplementary material

*Supplementary Table 1*: Environmental Microbiology Minimum Information (EMMI) for qPCR. *Supplementary Table 2*: Helminth target regions, primer sequences, and probe sequences for DNA amplification. *Supplementary Table 3*: Raw data for all samples. The supplementary material of this article is available at https://www.parasite-journal.org/10.1051/parasite/2026039/olm.
