# Supplementary material for: Molecular surveillance of intestinal parasites and Acanthamoeba species in soils from outdoor built environments in rural northwestern Argentina
Source: Parasite. 2026 Jul 29;33:41. doi: 10.1051/parasite/2026039 (PMC13427259; doi:10.1051/parasite/2026039)
Supplement: Supplementary file 1 — Supplementary Table 1: Environmental Microbiology Minimum Information (EMMI) for qPCR. [file parasite-33-41-s1.pdf]

Supplemental Table 1: Environmental Microbiology Minimum Information (EMMI) for qPCR.

**Environmental Sampling**	**qPCR**
Up to 50 grams	Target genes (supplemental table 2)
Argentina<br>Solazuti, Orán Department, Salta Province	Hold stage 95°C, 20 sec; Amplification Denaturation 95°C, 1 sec; Annealing 60°C, 20 sec.
Stored at 4°C and DNA extracted within 1 month	2x TaqMan® Fast Advanced Master Mix (Applied Biosystems, Foster City, CA) 3.5 µl
Exogenous DNA was used as an internal control to validate the extraction method. All samples had the internal control detect via qPCR	2 µl of template
**Sample Treatment**	Primers were used at 900 nM (Thermofisher)<br>Probe was used at 100 nM (Thermofisher)
Soil washed with PBS and 0.05% Tween 20	Chia Portable Real-time PCR (Chia Bio, Santa Clara, CA)<br>Or QS7 Pro Fast Real-time PCR System (Applied Biosystems, Waltham, Massachusetts, USA)
Flotation with 533 mg/ml sucrose solution (Specific Gravity 1.3)	2 µl of PCR-water was used as a negative control
**Sample Reduction**	Plasmids containing target parasite gene sequences was used as positive control
Samples are concentrated by a factor of 500	An exogenous DNA internal control was tested and all samples tested positive for the internal control Ct < 36.0
**Nucleic Acid Extraction**	**Analysis - qPCR**
MP fastDNA Spin kits for soil	Positive control standard curves were performed in duplicate
DNA eluent is 100 µl and stored at -20°C	Samples were tested in single
	All positive controls were compared to a set of known Ct values and were all within 5% range
	Lowest standard measured was approximately 0.019 fg/µl per kg of soil
	Automatic baseline and a threshold of 0.40 was used for all parasites
