# Supplementary material for: Molecular surveillance of intestinal parasites and Acanthamoeba species in soils from outdoor built environments in rural northwestern Argentina
Source: Parasite. 2026 Jul 29;33:41. doi: 10.1051/parasite/2026039 (PMC13427259; doi:10.1051/parasite/2026039)
Supplement: Supplementary file 2 — Supplementary Table 2: Helminth target regions, primer sequences, and probe sequences for DNA amplification. [file parasite-33-41-s2.pdf]

Supplemental Table 2: Helminth target regions, primer sequences, and probe sequences helminths for DNA amplification.

Parasite	Target region	Forward primer sequence (5' to 3')<br>Reverse primer sequence (5' to 3')<br>Probe sequence (5'FAM to 3')
Acanthamoeba species	18S rRNA	CCCAGATCGTTTACCGTGAA<br>TAAATATTAATGCCCCCAACTATCC<br>CTGCCACCGAATACATTAGCATGG
Ancylostoma species	ITS-2	GAATGACAGCAAACTCGTTGTTG<br>ATACTAGCCACTGCCGAAACGT<br>ATCGTTTACCGACTTTAG
Ascaris lumbricoides	ITS-1	TGCACATAAGTACTATTTGCGCGTAT<br>CCGCCGACTGCTATTACATCA<br>GAGCCACATAGTAAATT
Cryptosporidium species	DNA-J like protein	AACTTCACGTGTGTTTGCCAAT<br>CCAATCACAGAATCATCAGAATCG<br>CATATGAAGTTATAGGGATACCAG

Blastocystis species.	16s rRNA	AGTAGTCATACGCTCGTCTCAAA<br>TCTTCGTTACCCGTTACTGC<br>CGTGTAAATCTTACCATTTAGAGGA
Entamoeba histolytica	18S rRNA	GTTTGTATTAGTACAAAATGGCCAATTC<br>TCGTGGCATCCTAACTCACTTAGA<br>CAATGAATTGAGAAATGACA
Giardia intestinalis	16S rRNA	CATGCATGCCCGCTCA<br>AGCGGTGTCCGGCTAGC<br>AGGACAACGGTTGCAC
Necator americanus	ITS-2	CTGTTTGTCGAACGGTACTTGC<br>ATAACAGCGTGCACATGTTGC<br>CTGTACTACGCATTGTATAC
Strongyloides stercoralis	18s rRNA	GAATTCCAAGTAAACGTAAGTCATTAGC

		TGCCTCTGGATATTGCTCAGTTC<br>ACACACCGGCCGTCGCTGC
Toxocara canis	ITS-2	GCGCCAATTTATGGAATGTGAT<br>GAGCAAACGACAGCSATTTCTT<br>CCATTACCACACCAGCATAGCTCAC CGA
Toxocara cati	ITS-2	ACGCGTACGTATGGAATGTGCT<br>GAGCAAACGACAGCSATTTCTT<br>TCTTTCGCAACGTGCATTCGGTGA
Trichuris trichiura	ITS-1	TCCGAACGGCGGATCA<br>CTCGAGTGTCACGTCGTCCTT<br>TTGGCTCGTAGGTCGTT
Internal Amplification Control (IAC)	Synthetic sequence	CTAACCTTCGTGATGAGCAATCG<br>GATCAGCTACGTGAGGTCCTAC<br>TCGATGCACTCCAGTCCTCCT

*ITS = internal transcribed spacer; rRNA = ribosomal RNA
