# Supplementary material for: Molecular surveillance of intestinal parasites and Acanthamoeba species in soils from outdoor built environments in rural northwestern Argentina
Source: Parasite. 2026 Jul 29;33:41. doi: 10.1051/parasite/2026039 (PMC13427259; doi:10.1051/parasite/2026039)
Supplement: Supplementary file 3 — Supplementary Table 3: Raw data for all samples. [file parasite-33-41-s3.pdf]

Supplemental Table 3: Raw data of all samples.

House	Sample	Section	Ascaris Ct	Ascaris fg/µl per kg soil	Strongyloides Ct	Strongyloides fg/µl per kg soil	Toxcara canis Ct	Toxocara canis fg/µl per kg soil	Trichuris Ct	Trichuris trichiura fg/µl per kg soil	Acanthamoeba Ct	Acanthamoeba fg/µl per kg soil	Blastocystis Ct	Blastocystis fg/µl per kg soil	Giardia Ct	Giardia intestinalis fg/µl per kg soil
1	1-1	Entry									34.4	9092.569446				
1	1-2	Patio							33.3	25.92515335	35.6	1250.02671				
1	1-3	Latrine									34.6	3854.81176				
1	1-4	Patio									31.5	22825.43311				
2	2-1	Entry														
2	2-2	Latrine									35.6	1341.880213				
2	2-3	Patio														
2	2-4	Patio									36.2	872.2776447				
3	3-1	Entry									35.0	1140.297885			35	0.125435261
3	3-2	Patio									33.4	5041.437251				
3	3-3	Latrine									34.5	2461.081706				
3	3-4	Patio									37.2	438.0508926				
4	4-1	Patio							32.9	29.30333181	35.7	1088.82955				
4	4-2	Patio									34.0	3804.198531				
4	4-3	Patio									36.8	705.8372238				
4	4-4	Latrine														
5	5-1	Entry									35.2	1178.23426				
5	5-2	Latrine									35.2	1206.908262				
5	5-3	Patio									37.6	251.566256				
5	5-4	Patio									36.4	667.5235453				
6	6-1	Entry									31.7	25096.79768				
6	6-2	Latrine										xxxxxxxxx				
6	6-3	Patio									34.5	2562.589553				
6	6-4	Patio									32.0	18262.96438				
7	7-1	Entry									35.1	1478.352152				
7	7-2	Patio									31.7	12394.89294				
7	7-3	Latrine														
7	7-4	Patio														
8	8-1	Entry									36.5	710.0254651				
8	8-2	Patio										xxxxxxxxx				
8	8-3	Latrine														
8	8-4	Patio														
9	9-1	Entry					34.3	300.8487574			34.6	2397.750823				
9	9-2	Patio									33.3	5215.62204				
9	9-3	Latrine									35.7	783.3578064				
9	9-4	Patio														
10	10-1	Entry									33.2	6676.335867				
10	10-2	Latrine									35.3	1231.090518				
10	10-3	Patio									35.1	1608.510107				
10	10-4	Patio									36.1	701.7919509				
11	11-1	Entry														
11	11-2	Patio														
11	11-3	Latrine														
11	11-4	Patio									35.4	1047.267144				
12	12-1	Entry			31	0.215042242					32.8	4834.577134				
12	12-2	Patio			26.5	7.415641127					32.5	8997.26449				
12	12-3	Latrine											34.3	0.159068263		
12	12-4	Patio														
13	13-1	Entry									33.8	4152.856084				
13	13-2	Patio														
13	13-3	Latrine									36.6	590.4189661				
13	13-4	Patio									30.7	43733.02442				
14	14-1	Entry									36.0	740.8281944				
14	14-2	Patio									35.4	1603.680468				
14	14-3	Patio									36.8	482.8748772				
14	14-4	Latrine														
15	15-1	Entry									32.7	7795.523216				
15	15-2	Patio														
15	15-3	Latrine									33.7	4456.840511				
15	15-4	Patio					21.8	2188280.307			30.9	28479.59057				
16	16-1	Entry									35.3	1339.25449				
16	16-2	Patio									33.2	5863.97253				
16	16-3	Latrine														
16	16-4	Patio									31.6	26917.23038				
17	17-1	Entry									34.4	2825.570988				
17	17-2	Patio														
17	17-3	Latrine									33.2	4566.084283				
17	17-4	Patio									33.5	7173.114896				
18	18-1	Entry					28.3	23851.22323			35.9	1224.757463				
18	18-2	Patio									31.0	29704.46308				
18	18-3	Latrine									33.1	7054.990718				
18	18-4	Patio									37.1	542.5489443				
19	19-1	Entry									35.8	1043.699586				
19	19-2	Patio										xxxxxxxxx				
19	19-3	Latrine										xxxxxxxxx				
19	19-4	Patio									34.7	2522.464784				
20	20-1	Entry									34.1	3918.834063				
20	20-2	Latrine									33.5	4783.749532			33.9	33.02401034
20	20-3	Patio									37.0	488.5123619				
20	20-4	Patio									31.1	33546.43759				
22	22-1	Entry	23.5	1494.876596			26.4	76828.90199			36.9	947.6911107				
22	22-2	Latrine									34.5	7759.07235				
22	22-3	Patio	30.5	11.37852405							35.4	3277.31506				
22	22-4	Patio	31.5	9.356670312							33.5	91716.38417			32.9	1.39424961
23	23-1	Patio														
23	23-2	Entry														
24	24-1	Entry														
24	24-2	Latrine							34.6	99.72818257						
24	24-3	Patio	32.8	3.29366936												
24	24-4	Patio	36.1	0.338568401							33.3	5966.078046				
25	25-1	Entry	37.4	0.125695813							29.6	80694.40761				
25	25-2	Patio	38	0.099063683	36.5	0.019383502					32.4	10870.23632				
25	25-3	Latrine	33.7	1.307278249			26.2	63694.67648			34.1	2668.047875				
25	25-4	Patio	25.3	346.6639427	35.4	0.021850756			36.7	40.56377885	30.0	33702.17565				
25	25-5	Patio	29.6	28.40723255					34.4	130.1502121						
26	26-1	Entry	28.9	346.6639427												
26	26-2	Patio									29.9	44968.14995				
26	26-3	Latrine									35.6	1103.82688				
26	26-4	Patio									31.0	3373.946397				
28	28-1	Entry									35.9	704.5241321				
28	28-2	Patio														
28	28-3	Patio									37.5	302.7621961				
28	28-4	Latrine									34.5	2876.408764				
30	30-1	Entry									37.4	310.1237397				
30	30-2	Latrine								xxxx		xxxxxxxxx				
30	30-3	Patio								xxxx		xxxxxxxxx				
30	30-4	Patio								xxxx		xxxxxxxxx				
